# An arms‐race against resistance: leukemic stem cells and lineage plasticity

**DOI:** 10.1002/1878-0261.13606

**Published:** 2024-02-20

**Authors:** Alexander Waclawiczek, Aino‐Maija Leppä, Simon Renders, Andreas Trumpp

**Affiliations:** ^1^ Division of Stem Cells and Cancer German Cancer Research Center (DKFZ) and DKFZ‐ZMBH Alliance Heidelberg Germany; ^2^ Heidelberg Institute for Stem Cell Technology and Experimental Medicine (HI‐STEM gGmbH) Germany; ^3^ Department of Internal Medicine V, Hematology, Oncology and Rheumatology Heidelberg University Hospital Germany; ^4^ German Cancer Consortium (DKTK) Heidelberg Germany

**Keywords:** AML, lineage differentiation, LSC, plasticity, resistance, venetoclax

## Abstract

Acute myeloid leukemia (AML) therapy is undergoing rapid development, but primary and acquired resistance to therapy complicates the prospect of a durable cure. Recent functional and single‐cell multi‐omics approaches have greatly expanded our knowledge of the diversity of lineage trajectories in AML settings. AML cells range from undifferentiated stem‐like cells to more differentiated myeloid or megakaryocyte/erythroid cells. Current clinically relevant drugs predominantly target the myeloid progenitor lineage, while monocyte‐ or stem cell‐like states can evade current AML treatment and may be targeted in the future with lineage‐specific inhibitors. The extent of aberrant lineage plasticity upon therapeutic pressure in AML cells in conjunction with hijacking of normal differentiation pathways is still a poorly understood topic. Insights into the mechanisms of lineage plasticity of AML stem cells could identify both therapy‐specific and cross‐drug resistance pathways and reveal novel strategies to overcome them.

## Graphical abstract

AML therapy is undergoing a rapid evolution but therapy resistance hampers the hope for a true cure. Recent works have highlighted diverse lineage trajectories in AML at diagnosis and their plasticity upon relapse. Drugs often only target one lineage state providing an escape mechanism which needs to be counteracted in a personalized fashion using lineage‐specific inhibitors.

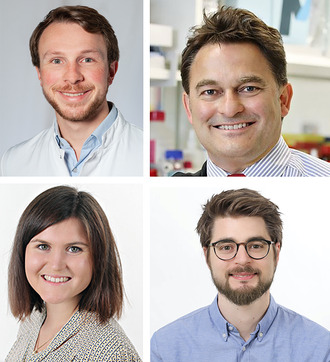

**Abbreviations**
5‐AZA: 5‐azacitidine
AML: acute myeloid leukemia
BCL‐2: B‐cell lymphoma‐2
BCL‐xL: B‐cell lymphoma‐extra large
DNA: deoxyribonucleic acid
FLAG‐IDA: fludarabine, aracytidine, G‐CSF, idarubicine
FLT3‐ITD: Fms like tyrosine kinase 3‐internal tandem domains
HSC: hematopoietic stem cell
HSCT: hematopoietic stem cell transplantation
IDH1/2: isocitrate dehydrogenase 1/2
KAT6A: lysine acetyltransferase 6A
KMT2A: histone‐lysine N‐methyltransferase 2A
LSC: leukemia stem cell
MAC‐Score: mediators of apoptosis combinatorial‐score
MCL1: induced myeloid leukemia cell differentiation protein‐1
mRNA: messenger ribonucleic acid
TP53: tumor protein 53
VEN: Venetoclax

The hierarchically organized hematopoietic system is headed by self‐renewing hematopoietic stem cells (HSCs). These cells give rise to multilineage and subsequently lineage‐restricted progenitor cells followed by maturation into effector cells. In the healthy setting, the gradient of self‐renewal potential is inversely proportional to the degree of differentiation. Transformation of healthy stem or progenitor cells can give rise to a range of pre‐malignant or malignant precursor states for example clonal hematopoiesis or myeloproliferative neoplasms (MPN). Although these can proceed towards genetically heterogeneous acute myeloid leukemia (AML), AML may also arise by other sometimes complex genetic alterations. AML is the most frequent form of acute leukemia in adults and linked to dismal prognosis. This is particularly true in elderly patients not eligible for highly toxic chemotherapy combinations [e.g. Fludarabine, Cytarabine, G‐CSF, Idarubicin (FLAG‐IDA)] followed by allogenic hematopoietic stem cell transplantation (HSCT). After decades of unsuccessful attempts to significantly improve AML therapies in elderly patients, several breakthrough drugs are now finding their way into clinical practice. These include targeted therapies against gain‐of‐function oncoproteins IDH1/2 and FLT3‐ITD or more widely applicable inhibitors against the pro‐survival factor BCL‐2 or the epigenetic scaffold protein Menin. Although these novel therapies show clear clinical efficacy, they have not been proven to be curative without consolidating HSCT, and the development of resistance (often pan‐resistance) is an almost inevitable clinical roadblock [1, 2].

In AML, response to therapy is driven by the heterogeneous population of disease‐propagating leukemic stem cells (LSCs) [3]. These cells show different degrees of inherent treatment resistance and may acquire secondary resistance during treatment through genetic and non‐genetic mechanisms. Historically, therapy resistance in AML has largely been attributed to genetic mechanisms as emphasized by the AML risk classification that is based on genomic traits [4]. However, most treatment resistance cannot be explained by mutations alone but is due to a complex combination of genetic and non‐genetic processes. Under the influence of therapy pressure, cell states are dynamically acquired with the consequence that survival and growth are no longer dependent on the given therapy target protein. As these states are often reversible and can change again under alternative therapies, evolution will select leukemic cells with highest potential for plasticity, namely LSCs. Genetic events, especially gain‐of‐function mutations, can be effectively targeted using inhibitors. In contrast, plasticity‐induced resistance of cancer cells poses a greater challenge in identifying dependencies that would counteract the growth and survival of leukemia.

In MPNs, it has been well established that mutations generate lineage‐primed diseases with increased production of semi‐functional mature cells originating from a mutated stem or progenitor cell. For example, in chronic myelomonocytic leukemia, *TET2* mutations are often seen with monocytosis while *MPL* mutations in essential thrombocythemia lead to an increased number of altered platelets. In AML, a small number of mutations have also been associated with differentiation stages. For example, mutations of the RAS pathway often bias AML towards a monocytic trajectory while the presence of a PML‐RAR translocation is associated with a promyelocytic state. Importantly, recent work by several groups has shown that certain differentiation states in AML can be substantial drivers of drug response or resistance. By using deconvolution tools to generate lineage maps from bulk transcriptome data of AML patients, these studies have shown that patient response to an extensive *ex vivo* array of drugs is predominantly governed by the differentiation state of the AML [5, 6]. Importantly, the vast majority of clinically used inhibitors target early myeloid progenitors whereas HSC‐like leukemia populations are predominantly resistant, providing a possible explanation for pan‐drug resistance due to resemblance to an undifferentiated plastic state. This suggests that AML cells may gain the molecular machinery to resist therapy by hijacking lineage differentiation programs found in healthy hematopoiesis or dedifferentiate to gain plasticity.

It is crucial to our understanding of therapy resistance to address lineage differentiation and plasticity in AML. Beyond the undifferentiated or myelomonocytic lineage spectrum, it is well established that additional lineage categories of AML exist. These include the rare erythroblastic, megakaryoblastic, and mixed‐lineage leukemia, most of which are so far not considered in therapeutic decision making. However, two recent studies linking single‐cell transcriptomic and genomic DNA profiling have uncovered the surprising extent of lineage‐committed leukemia cells in individual AML patients [7, 8]. Mutation‐bearing AML cells can not only be found in myeloid lineages but also in the naive stem cell compartment and in trajectories phenocopying erythroid and megakaryocyte lineages [7]. Multilineage blasts are particularly pronounced in TP53 mutated AMLs, which are associated with poor clinical response to both chemo‐ and Venetoclax/5‐Azacytidine (VEN/AZA)‐therapies [8]. However, differentiation and self‐renewal must be tightly balanced to avoid loss of LSC‐potential while retaining lineage‐specific features that convey therapy resistance [3]. Future studies will need to address how this balance is maintained in AML, in particular with regards to lineage plasticity.

Several well‐studied mechanisms of AML therapy resistance like cell cycle states [9], cellular metabolism [10], and epigenetic dependencies [11] are also essential parts of lineage differentiation in healthy hematopoiesis. This also includes anti‐apoptotic dependencies. For example, BCL‐2 inhibition by VEN targets  healthy granulopoiesis [1] while cells in the megakaryocytic‐ and erythropoietic lineages mainly depend on BCL‐xL [12]. Along these lines, we have recently shown that prediction of VEN/AZA response is determined by the combined presence of BCL‐2, BCL‐xL, and MCL‐1 in LSCs which can be quantified by the mediators of apoptosis combinatorial‐score (MAC‐Score) [3]. However, it remains unclear what determines the ratio of BCL‐2, BCL‐xL, and MCL‐1 in disease driving LSCs. Several mechanisms may affect this, with the identity and order of disease driving mutations in combination with the cell‐of‐leukemia origin likely presenting important factors. While these factors are apparently different in each patient and difficult to retrospectively analyze, we hypothesize that their expression is mediated by the differentiation state of LSCs in each patient. The therapeutic pressure triggered by therapies such as VEN or BCL‐xL inhibitors may, however, lead to induction of an active plasticity mode. New differentiation states may then be selected for, in which the dependence of the inhibited pro‐survival protein (BCL‐2 or BCL‐xL) is released and naturally replaced by another family member with similar function. This could lead to upfront or secondary resistance of LSCs to such drugs by acquiring a particular insensitive differentiation state while maintaining self‐renewal. Future work may address these hypotheses and allow the design of strategies that add a block to such differentiation pathways leading to therapy escape.

The extent to which therapeutic pressure can drive AML cells to enter certain lineage trajectories to escape elimination is the subject of several current studies. In a yet undefined proportion of patients, a newly identified rare monocytic subpopulation with stem cell features has been shown to be positively selected in response to 5‐AZA/VEN therapy pressure and may also be associated with relapse [13]. KMT2A‐mutated AML has been shown to develop resistance to Menin inhibitors by transitioning into a monocytic state in which KMT2A binding to chromatin is mediated through KAT6A instead of Menin, allowing drug escape [14]. These studies provide strong evidence that lineage plasticity in combination with Darwinian selection can be relevant for therapy response in AML. Nevertheless, larger unbiased studies of paired diagnosis‐relapse samples are required to better understand the full‐scale implications of therapy escape and resistance of AML cells by entering certain differentiation trajectories. Paired analysis of pediatric AMLs has uncovered that AML HSPCs are epigenetically rewired to divert from the myeloid lineage into a more undefined, plastic lineage state upon relapse. Although the diversion was seen in AMLs with diverse mutational backgrounds, the underlying molecular drivers point towards multiple, mutation‐specific mechanisms [11]. These data show that de‐differentiation can also be used for therapy escape, a phenomenon often observed in solid tumors and metastasis.

In conclusion, employment of either healthy lineage trajectories or de‐differentiation programs by leukemia (stem) cells in response to therapy stress may mediate therapy escape by plasticity without the necessity of additional mutations (Fig. 1). Widely considered side‐effects like anemia, neutropenia or thrombocytopenia might be signs of a drug's lineage specificity and could be utilized as personalized therapies depending on the patient's AML lineage trajectories. Drug developers need to consider lineage plasticity to boost the arsenal against a wider range of AML lineage stages and clinicians need to develop tools to continuously identify them during treatment.

**Fig. 1:**
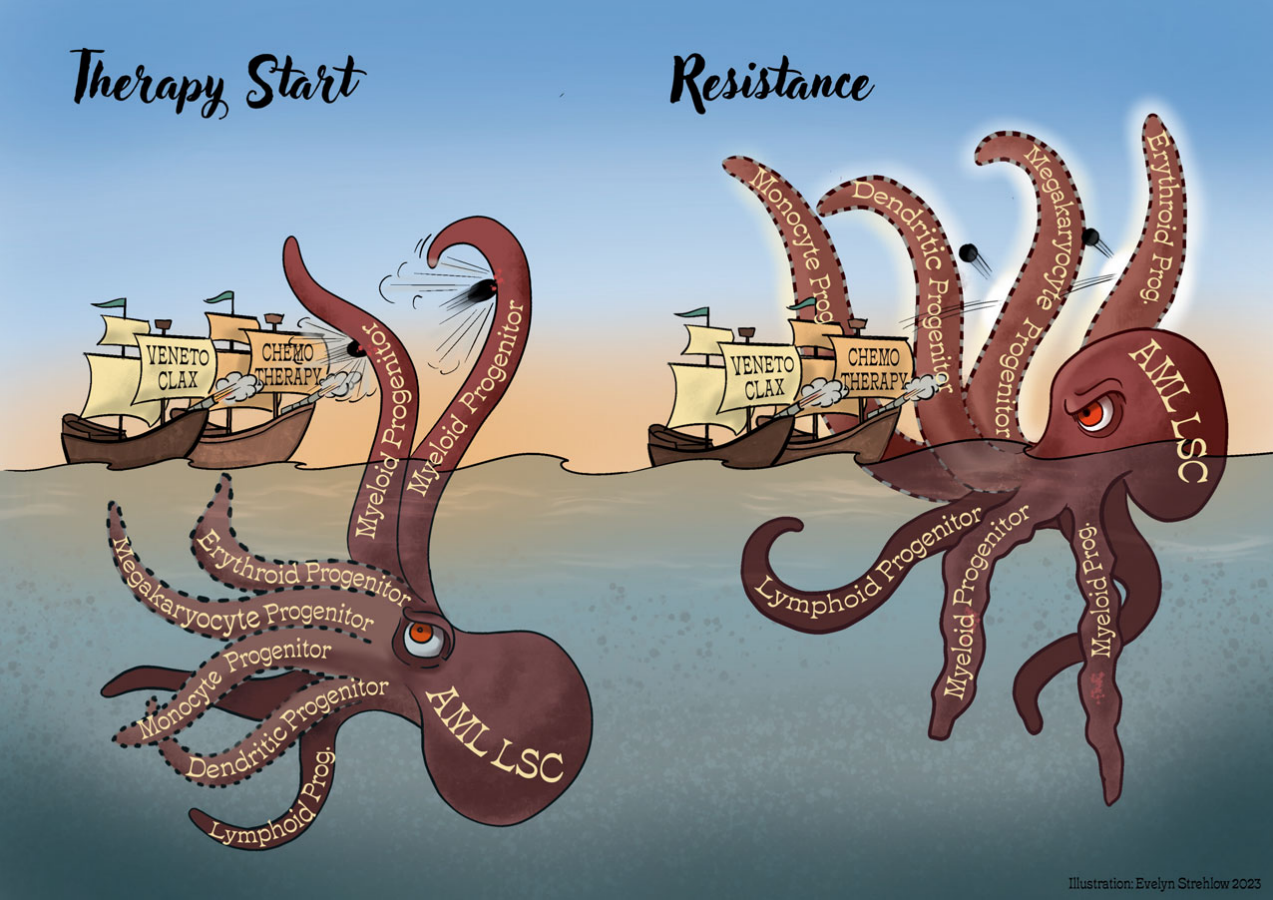
Clinically relevant therapies show a target bias that can be exploited by AML LSCs via lineage plasticity to achieve resistance.

## Author contributions

AW wrote the first draft, A‐ML, SR, AW, and AT revised and optimized the manuscript. AW and AT designed the figure.

## Conflict of interest

The authors declare no conflict of interest.
